# Interplay of Transcriptomic Regulation, Microbiota, and Signaling Pathways in Lung and Gut Inflammation-Induced Tumorigenesis

**DOI:** 10.3390/cells14010001

**Published:** 2024-12-24

**Authors:** Beatriz Andrea Otálora-Otálora, César Payán-Gómez, Juan Javier López-Rivera, Natalia Belén Pedroza-Aconcha, Sally Lorena Arboleda-Mojica, Claudia Aristizábal-Guzmán, Mario Arturo Isaza-Ruget, Carlos Arturo Álvarez-Moreno

**Affiliations:** 1 Grupo de Investigación INPAC, Unidad de Investigación, Fundación Universitaria Sanitas, Bogotá 110131, Colombia; claristizabal@unisanitas.edu.co; 2 Dirección Académica, Universidad Nacional de Colombia, Sede de La Paz, La Paz 202017, Colombia; cepayang@unal.edu.co (C.P.-G.); npedroza@unal.edu.co (N.B.P.-A.); 3 Grupo de Investigación INPAC, Specialized Laboratory, Clinica Universitaria Colombia, Clínica Colsanitas S.A., Bogotá 111321, Colombia; jjlopez@colsanitas.com; 4 Grupo de Investigación INPAC, Facultad de Medicina, Fundación Universitaria Sanitas, Bogotá 110131, Colombia; slarboledamo@unisanitas.edu.co; 5 Keralty, Sanitas International Organization, Grupo de Investigación INPAC, Fundación Universitaria Sanitas, Bogotá 110131, Colombia; misaza@unisanitas.edu.co; 6 Infectious Diseases Department, Clinica Universitaria Colombia, Clínica Colsanitas S.A., Bogotá 111321, Colombia; calvarez@colsanitas.com

**Keywords:** inflammation-induced tumorigenesis models, transcription factors, long non-coding RNAs, transcriptional regulatory network, inflammatory-related cell membrane receptors, microbiota, signaling pathways, oral–gut–lung axis

## Abstract

Inflammation can positively and negatively affect tumorigenesis based on the duration, scope, and sequence of related events through the regulation of signaling pathways. A transcriptomic analysis of five pulmonary arterial hypertension, twelve Crohn’s disease, and twelve ulcerative colitis high throughput sequencing datasets using R language specialized libraries and gene enrichment analyses identified a regulatory network in each inflammatory disease. IRF9 and LINC01089 in pulmonary arterial hypertension are related to the regulation of signaling pathways like MAPK, NOTCH, human papillomavirus, and hepatitis c infection. ZNF91 and TP53TG1 in Crohn’s disease are related to the regulation of PPAR, MAPK, and metabolic signaling pathways. ZNF91, VDR, DLEU1, SATB2-AS1, and TP53TG1 in ulcerative colitis are related to the regulation of PPAR, AMPK, and metabolic signaling pathways. The activation of the transcriptomic network and signaling pathways might be related to the interaction of the characteristic microbiota of the inflammatory disease, with the lung and gut cell receptors present in membrane rafts and complexes. The transcriptomic analysis highlights the impact of several coding and non-coding RNAs, suggesting their relationship with the unlocking of cell phenotypic plasticity for the acquisition of the hallmarks of cancer during lung and gut cell adaptation to inflammatory phenotypes.

## 1. Introduction

The interplay between transcriptomic regulation, the microbiota, and signaling pathways is crucial in understanding the development of lung and gut inflammation-induced tumorigenesis. Inflammation can positively and negatively affect the establishment of tumorigenesis based on the duration, scope, and sequence of related events through the regulation of signaling pathways involved in cell differentiation, proliferation, survival, migration, and angiogenesis, regulated by growth factors, pro-inflammatory cytokines, and proangiogenic factors [1]. Chronic inflammation caused by persistent infections and metabolic disorders could be related to the increased cancer risk and accelerated cancer progression [2]. Tumor tissue is full of microbial (bacteria, fungi, and viruses) DNA, RNA, peptides, cell wall components, and metabolites, like fatty acids and inosine, which can be build up inside tumors and bind to membrane receptors on inflammatory, immune, and cancer-related cells [3]. The microbiota composition in terms of quality, quantity, types, and localization in the oral–gut–lung axis is influenced by genetics, epigenetics, the immune system, diet, antibiotics, and medication consumption, along with other environmental factors in health and disease [4]. Furthermore, the microbiota is metabolically active, producing toxins with beneficial effects that destroy pathogenic strains, altering the pH of the local environment, metabolizing key nutrients to starve competitors, as well as maintaining mucosal layers and epithelial integrity [5]. Commensal and pathogenic microbes are critical regulators of the host immune system and inflammation; consequently, they have the potential power to impact tumor establishment and progression through chronic activation of inflammation, alteration of the tumor microenvironment, induction of genotoxic responses, as well as the regulation of inflammatory and cancer-related cell signaling pathways and metabolism [6]. Therefore, in the lung and gut cell microenvironment, microbes can activate, inhibit, or withstand the tumor immune microenvironment through the presentation of microbial antigens by cancer, inflammatory, and immune cells, microbial antigen mimicry shared with tumor antigens, microbe-induced immunogenic cell death, microbial adjuvanticity mediated by pattern recognition receptors, microbe-derived metabolites, and microbial stimulation of inhibitory checkpoints [7].

Pulmonary arterial hypertension (PAH), Crohn’s disease (CD), and ulcerative colitis (UC) are chronic inflammatory conditions that are often studied as models for inflammation-induced tumorigenesis because they exhibit key characteristics that link chronic inflammation to cancer development. Accumulation of genetic and epigenetic mutations in tumor suppressor genes and oncogenes, including deregulated expression of coding and non-coding RNAs, aberrant DNA methylation and histone modifications, parallel those seen in lung, gastric, and colon environments, and can be rapidly acquired in the inflammatory context of PAH, CD, and UC, contributing to the malignant transformation of epithelial cells [8,9]. A lung’s chronic inflammatory state also promotes genetic and epigenetic changes that alter gene expression in intestinal epithelial cells [10], which might increase the risk of gastrointestinal cancers [11]. PAH is a malignant pulmonary vascular remodeling disease characterized by perivascular inflammation and an increase in pulmonary artery pressure, resulting in right heart failure, with pharmacological treatments capable of dilating pulmonary arterials, improving the quality of life and survival of patients [12]. The morbidity and mortality of inflammatory lung disease are still high, and the hallmark of many respiratory diseases is inflammation [13]. PAH and lung cancer have many risk factors in common, such as smoking, environmental toxins, and systemic inflammatory diseases like scleroderma, suggesting a shared etiological basis [14]. Some case studies have reported an increased risk of lung cancer in patients with PAH, suggesting a potential indirect link, as within the 10-year observation period, the incidence of cancer is significantly higher in PAH patients, suggesting an important comorbidity that might induce the application of specific screening programs [15]. PAH shows a great complexity comparable to lung cancer, evidenced by the significant number of differentially expressed genes (DEGs), like those observed in lung cancer, which might be related to the difficulty in treating PAH [16]. Lung cancer has DEGs and transcription factors (TFs) in common with PAH, co-expressed in gene networks as regulators of the most frequently dysregulated DEGs, and with experimental evidence related to the acquisition of cancer stem-like characteristics and differentiation to cancer cells [10]. The hypothesis of establishment or “quasi-malignancy” suggests that lung cancer and severe PAH share several hallmarks of cancer [17]. In PAH, many cells in the vascular wall become abnormal, trying to survive under stress conditions such as inflammation and pseudo-hypoxia, mainly showing phenotypic, angiogenic, and glycolytic switches like tumor cells, related to the reduction of oxygen consumption, mitochondrial respiration, increased glycolytic metabolism [18], genome instability, mutations, inflammation, avoidance of immune destruction, and apoptosis [4,19]. Chronic inflammation and continuous vascular injury in the pulmonary arteries leads to persistent cellular stress, vascular remodeling, excessive tissue remodeling, fibrosis, and cellular hyperplasia, which involves hyperproliferation of pulmonary artery smooth muscle cells and endothelial cells, along with resistance to apoptosis and processes that resemble hallmarks of cancer, like uncontrolled cell growth and resistance to cell death, increasing vascular cells’ probability of undergoing further malignant transformation [10,20]. In PAH and cancer cells, mitochondrial metabolism induces a metabolic shift during vascular remodeling, associated with increased oxidative stress, generating reactive oxygen and nitrogen species in pulmonary endothelial and smooth muscle cells, causing DNA damage, genomic instability, mutations, and activation of oncogenic pathways, directly contributing to the initiation and progression of cancer [21]. Hypoxia is a hallmark of PAH, where impaired blood flow and oxygen delivery activate hypoxia-inducible factor 1-alpha signaling pathways, which regulates genes involved in angiogenesis, glycolysis, and cell survival, impairing immune surveillance by altering T-cell function, natural killer (NK) cells, and other immune effectors, allowing transformed cells to escape detection and grow unchecked, increasing the risk of lung cancer [22].

Inflammatory bowel disease (IBD) is an idiopathic intestinal inflammatory disease including CD and UC caused by long-term inflammation joined with immune dysregulation causing gastrointestinal tract damage [23] and is linked to the interaction of multiple factors, including environmental, genetic, intestinal microbiota, and immune response factors [24]. Chronic inflammation in CD and UC also generates recurrent epithelial injury and repair cycles that lead to dysplasia, which increases gastrointestinal cancer risk [25]. Case reports of CD patients with chronic inflammation in the upper gastrointestinal tract produce emerging evidence that promotes epithelial injury and metaplasia associated with gastric cancer [26]. UC patients have a 2% risk at 10 years, 8% risk at 20 years, and 18% risk at 30 years to develop colon cancer after UC diagnosis [27]. Gut chronic inflammation leads to prolonged immune activation, pro-inflammatory cytokine (e.g., IL-6, TNF-α, and IL-1β) and growth factor (e.g., VEGF and PDGF) release, and oxidative stress, which triggers cycles of tissue damage and repair, increasing the likelihood of errors during cell division, suppressing anti-tumor immune surveillance, and facilitating the survival of aberrant cells, which creates an enabling environment for cell proliferation, survival, and angiogenesis, increasing the risk of colon cancer [28].

Gut and airway microbiota can synthesize and secrete metabolites, which maintain general homeostasis, but imbalances between beneficial bacteria such as bacteroides and short-chain fatty acid (SCFA)-producing bacteria and potential pathogenic bacteria such as trimethylamine N-oxide (TMA/TMAO)-associated bacteria could be linked with the initiation and development of PAH [29]. Intestinal dysbiosis (microbial imbalance) exists in patients with PAH, mediating systemic inflammation or immunity via bacteria-related metabolites [12]. In UC and CD, there is an overactive immune response to microbial antigens, creating a microenvironment favorable for tumor initiation, as regular surveillance colonoscopies in IBD patients often detect inflammation and dysplastic lesions in colonic or gastric mucosa, colocalized with microbiota dysbiosis producing metabolites that might also promote DNA damage [30]. The inflamed gut and lung microenvironment is rich in tumorigenic factors, such as prostaglandins, matrix metalloproteinases, and microbial byproducts, that induce DNA damage [31]. The interplay of microbiota dysbiosis and chronic inflammatory diseases can create a tumor-promoting microenvironment [32]. The microbiota generates inflammatory-exacerbated responses by activating pattern recognition receptors, like Toll-like receptors, on immune cells, which promotes the continuous production of inflammatory cytokines and chemokines related to tissue damage and repair processes that favor tumorigenesis, while inflammatory cells secrete pro-inflammatory factors, generate reactive oxygen species and reactive nitrogen species capable of damaging DNA, proteins, and lipids and suppressing the adaptive immune response through regulatory T cells and myeloid-derived suppressor cells, which help tumors evade immune surveillance [33]. Inflammatory diseases continuously attract immune cells like macrophages, neutrophils, and lymphocytes, which can shift from anti-tumor to pro-tumor phenotypes under the influence of the tumor microenvironment [34]. Microbial metabolites, such as SCFAs, can modulate histone acetylation and DNA methylation, altering gene expression patterns to favor tumor development [35].

In our previous global transcriptomic analysis, the transcriptional regulatory networks (TRNs) of gastric, colon, and lung cancers were identified and analyzed under the regulation of the tumor microbiome network in the oral–gut–lung axis [36]. Cell receptors were highlighted between common upregulated DEGs that might be involved in the host–microbiome crosstalk and the control of specific deregulated signaling pathways in gastric, colon, and lung cancer-related cells. Now, we want to make a transcriptomic analysis of PAH and IBD, to identify key deregulated DEGs, TFs, and non-coding regulatory RNAs characteristic of these inflammatory diseases common to lung, gastric, and colon cancer, considering the key function of the microbiota for the activation of signaling pathways associated with these lung and gut inflammatory processes that might be involved in the acquisition of the hallmarks of cancer and the establishment of tumorigenic processes.

In this study, we aim to achieve the following: (1) Identify DEGs in gut and lung inflammatory diseases, highlighting the membrane receptors related to host cells and the microbiota crosstalk and key regulatory coding and non-coding RNAs that control the expression of the other DEGs in every type of inflammatory disease; and (2) perform a joint transcriptomic network analysis between gene ontology and regulatory and transcriptional network analysis, along with a profound review of the scientific data, to highlight the importance of the microbiota in the regulation of genomic (DNA mutations), transcriptomic (RNA expression), epigenomic (DNA methylation, non-coding RNAs, histone and protein modifications), proteomic, and metabolomic process stability. The specific coding and non-coding TRN and a GRN in inflammatory-related cells will be established, which in turn may control the interaction with the microbiome network to unlock phenotypic plasticity for the acquisition of the hallmarks of cancer as we previously did for gut and lung cancer [36], only now with the adaptation of the gut–lung axis cells to inflammatory processes. Hence, using PAH, CD, and UC as models for inflammation-induced tumorigenesis, the key regulatory RNAs will be identified in inflammatory TRNs that link with the microbiome network through specific membrane receptors to become attractive candidates that can be used as multiomics biomarkers for the development of specific diagnostic tools and treatments against gut and lung inflammatory diseases and the prevention of tumorigenic processes.

## 2. Materials and Methods

### 2.1. Data Selection and Construction of Coding and Non-Coding Transcriptomic Gene Regulatory Networks of Lung and Gut Inflammatory Diseases

We performed a search of the National Center for Biotechnology Information (NCBI) GEO database in Bethesda, MD, USA (http://www.ncbi.nlm.nih.gov/geo/) (accessed on 7 October 2024) to identify studies of global gene expression in human tissues affected by PAH, CD, and UC, as well as in healthy controls. For PAH, the data came from lung tissue, while for CD and UC, the data came from intestinal tissue. The main goal is to identify common DEGs in most of the datasets of every inflammatory disease, despite the variability present in the different datasets, represented by factors (e.g., age, gender, disease severity or stage, diet, and environment) that can specifically differentiate the populations analyzed in each dataset, since it is the same inflammatory disease diagnosed with specific characteristics at the clinical level and treated as such. To ensure the quality of the data, for Affymetrix datasets, we inspected the distributions of probe intensities to identify and address technical inconsistencies and performed principal component analysis (PCA) to evaluate sample clustering by experimental groups both before and after normalization. Quantile normalization was subsequently applied to eliminate technical variations and ensure comparability across datasets. This method ranks expression levels in an ascending matrix, calculates the mean for each rank, and adjusts each sample accordingly to produce comparable data. For RNA-seq datasets, we examined the distributions of raw counts and performed PCA to assess the alignment of samples with their expected experimental groups. To reduce noise and improve robustness, we filtered out genes with low counts or low variability across samples before normalization. Normalization methods appropriate for RNA-seq, such as those accounting for library size differences, were then applied to standardize the data. The datasets listed in Table 1 were selected to ensure an adequate and representative sample size for each condition. This included five PAH datasets, twelve for CD and twelve for UC, with a balance of normal and affected samples. DEGs were considered differentially expressed if the fold change was greater than 1.5 or less than −1.5 and the adjusted *p*-value was less than 0.05. Overregulated DEGs found in at least three PAH datasets, eight CD datasets, and nine UC datasets were selected as key genes for each inflammatory disease, highlighting the transcriptional regulators.

We used the ncFANs v2.0 platform to annotate coding and non-coding RNAs functionally and to construct a regulatory network for lung and intestinal inflammatory diseases [37]. This platform allowed us to analyze the relationship between long non-coding RNAs (lncRNAs) and protein-coding genes (PCGs) related to TFs. The resulting coding and non-coding transcriptional regulatory network (cncTRN) was analyzed for Gene Ontology (GO) and KEGG pathways and visualized using Cytoscape 3.10.2 [38]. GENIE3 (GEne Network Inference with Ensemble of trees) is the algorithm used for the inference of inflammatory disease GRNs based on variable selection with ensembles of regression trees, where the expression pattern of a target gene is predicted from the expression patterns of all the other genes (input genes), using tree-based ensemble methods like Random Forests or Extra-Trees [39]. The GRNs were constructed with the expression matrix of common overregulated genes, using the GSE113439 dataset for PAH, GSE75214 dataset for CD, and GSE92415 for UC, and R library BioNERO version 1.12.0, an all-in-one R/Bioconductor package for comprehensive and easy biological network reconstruction [40].

### 2.2. Transcriptomic Regulatory Network Analysis of Lung and Gut Inflammatory Diseases

DAVID’s annotation analysis of highly expressed DEGs of membrane receptors, TFs, and lncRNA identified signaling pathways associated with the interaction of inflammatory-related cells and microorganisms in each type of inflammatory disease that might be related to the acquisition of the hallmarks of cancer [41], and ToppFun analysis made a functional enrichment based on transcriptomes, regulomes (TFBS and lncRNA), ontologies (GO, Pathway), phenotypes (human disease), pharmacomes (Drug-Gene associations), and literature co-citations [42]. The results section focuses on analysis of every inflammatory disease, highlighting the key function of main coding and non-coding regulators in signaling pathways regulated by the microbiota and membrane receptor crosstalk located in membrane rafts and receptor complexes, the regulation of genomic and epigenomic mechanisms of gene expression, biological functions involved in the acquisition of the hallmarks of cancer, and the comparison between the DEGs and TFs shared by gut and lung inflammatory diseases and cancer types that might be related to the acquisition of the hallmark of cancer. PAH, CD, and UC cncTRN characterize the complex gene regulatory programs involved in inflammation that might be essential to unlock the phenotypic plasticity of gut and lung cells thorough the regulation of expression in a specific spatial, temporal, and sequential way, probably according to microbiome and host transcriptomic network crosstalk [36]. PAH, CD, and UC GRN identified the target genes of each transcriptional regulator. In the discussion, BioRender © 2024 images sum up the knowledge in the field of the role of microbiota in lung and gut inflammation-induced tumorigenesis. The first section analyzes the contribution of coding and non-coding transcriptional regulators of gut and lung inflammatory diseases to tumorigenesis. The second section analyzes the host-microbiota crosstalk through lung and gut cell receptors present in membrane rafts and complexes, highlighting the known microorganisms that might be involved in the activation of the key signaling pathways identified, the downstream unique transcriptomic changes, and their relative contribution to the inflammatory and tumor disease pathogenesis. The third section analyzes the transcriptomic association between gut and lung inflammatory and tumorigenic diseases, highlighting the TFs and biological processes that might be involved in the acquisition of the hallmarks of cancer. The fourth section analyzes the interactions between transcriptomic regulation and microbiota within inflammation and tumorigenesis under previous studies and multiomics approaches, as well as future directions in the field. In the conclusion, we summarize for every inflammatory disease, the key coding and non-coding regulators, membrane receptors that might interact with microorganisms, and signaling pathways with experimental evidence that might be activated, to establish the current state of the art in microbiome and establishment of inflammatory and tumorigenic disease research as the guiding core and starting point for future multiomics studies in this field.

## 3. Results

**Figure 1:**
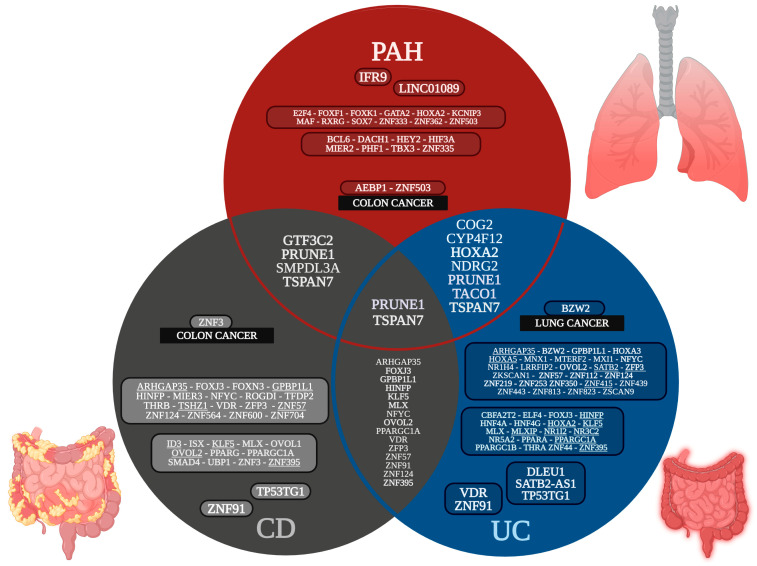
Venn diagram with the transcriptomic metafirm in common and unique to each type of inflammatory disease. Created with BioRender.com.

PAH has 250 common DEGs upregulated in at least three datasets, of which 24 are TFs (Table S1.1); CD has 435 common upregulated DEGs in at least eight datasets, of which 31 are TFs (Table S1.4); and UC has 522 common upregulated genes in at least nine datasets, of which 48 are TFs (Table S1.7). There are two common upregulated DEGs between PAH, CD, and UC (Figure 1). Prune exopolyphosphatase 1 (PRUNE1) is related to epigenetic reprograming through acetylation and metabolic pathways, and Tetraspanin 7 (TSPAN7) is in transmembrane helix. There are two more (GTF3C2, and SMPDL3A) common upregulated DEGs between PAH and CD. GTF3C2 is in the nucleoplasm along fifteen TFs, while SMPDL3A is in extracellular exosomes. There are four more (COG2, CYP4F12, NDRG2, and TACO1) common upregulated DEGs and one TF (HOXA2) between PAH and UC. COG2 is in the trans-golgi network membrane; CYP4F12 is in the endoplasmic reticulum membrane and transmembrane helix; NDRG2 is in the golgi apparatus, extracellular exosomes, transmembrane helix, and related to epigenetic reprograming through acetylation; TACO1 is in mitochondrion, related to primary mitochondrial disease; and HOXA2 is in the nucleoplasm along with 14 TFs and related to negative regulation of transcription by RNA polymerase II. There are 227 common upregulated DEGs between CD and UC, of which 15 are TFs, located in the mitochondrion and extracellular exosomes, related to metabolic pathways, epigenetic reprograming through acetylation, primary mitochondrial disease, fatty acid degradation, peroxisome proliferator-activated receptor (PPAR) and AMP-activated protein kinase (AMPK) signaling pathways, proteoglycans in cancer, and citrate cycles (TCA cycle).

### 3.1. Pulmonary Arterial Hypertension (PAH)

**Figure 2:**
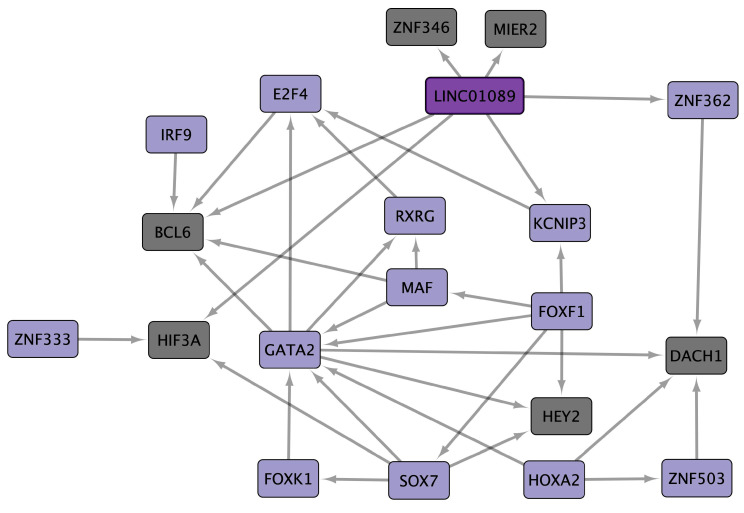
Coding (lilium and grey) and non-coding (purple) transcriptional regulatory network (cncTRN) of key upregulated transcription factors (TFs) and lncRNA in pulmonary arterial hypertension (PAH). Created with Cytoscape.

Interferon regulatory factor 9 (IRF9) is common in four PAH datasets, and twenty-three TFs and a lncRNA (long intergenic non-protein coding RNA 1089 (LINC01089)) are common in three PAH datasets. cncTRN analysis identified IRF9, LINC01089, and another twelve TFs as key regulators of PAH (Figure 2). IRF9 is also a key regulator according to PAH GRN, along with other 21 TFs, and regulates 189 PAH common overregulated DEGs, including LINC01089, which are co-expressed in lung cancer models and are related to cell development and differentiation, regulation of cellular component size, and cytoplasmic vesicle membrane and retromer complex binding (Table S1.3). Annotation analysis identified PAH TFs related to positive and negative regulation of transcription and form transcription regulator complexes. PAH upregulated DEGs are related to the function of the extracellular exosomes (AEBP1), plasma membrane (HIF3A and KCNIP3), transmembrane helix (GLMP), cytoplasmic vesicles, lysosome, membrane raft, golgi, endosome, phagosome, and endoplasmic reticulum. Upregulated DEGs are also involved in signaling pathways like mitogen-activated protein kinase (MAPK), neurogenic locus notch (NOTCH), human papillomavirus (HPV) and hepatitis C (HPC) infection (IRF9, and HEY2), gastric (HOXA2, and PHB1), non-small and small cell lung cancer (RXRG), chemical carcinogenesis-receptor activation (BCL6 and RXRG), and pathways in cancer (HEY2 and RXRG).

Upregulated DEGs and TFs are also involved in several cellular and biological processes that might be involved in the acquisition of the hallmarks of cancer, like angiogenesis, positive and negative regulation of cell proliferation, cell proliferation, cell migration, cell differentiation, cell motility, cell activation, immune response, apoptosis, and epigenetics reprogramming, like histone modification, histone deacetylase binding, epigenetic regulation of gene expression, and the formation of protein–DNA complexes (Table 2). PAH has twenty-one overregulated DEGs previously involved in cancer, including membrane receptors and TFs. According to our previous transcriptomic analysis [36], PAH and lung cancer have three (C1QBP, COX6B1, and SLC12A8) common overregulated genes located in the transmembrane helix related to negative regulation of transcription by RNA polymerase II and chemical carcinogenesis-reactive oxygen species. PAH and gastric cancer have two (CDC25B, and IFI6) common overregulated genes located in the nucleoplasm, along with fifteen TFs, the transmembrane helix, and the endoplasmic reticulum membrane, related to negative regulation of the apoptotic process. PAH and colon cancer have eight (CDC25B, GJA4, MXRA7, S100A11, SLC29A1, SLC3A2, TIMP3, and TRMT1) common overregulated genes located in the nucleoplasm, along with fifteen TFs, the transmembrane helix, lysosomal membrane, and extracellular exosomes, related to negative regulation of cell population proliferation, symbiont entry into host cells, as well as two common overregulated TFs (AEBP1, and ZNF503) located in extracellular exosomes, related to negative regulation of cell population proliferation and transcription.

### 3.2. Crohn’s Disease (CD)

**Figure 3:**
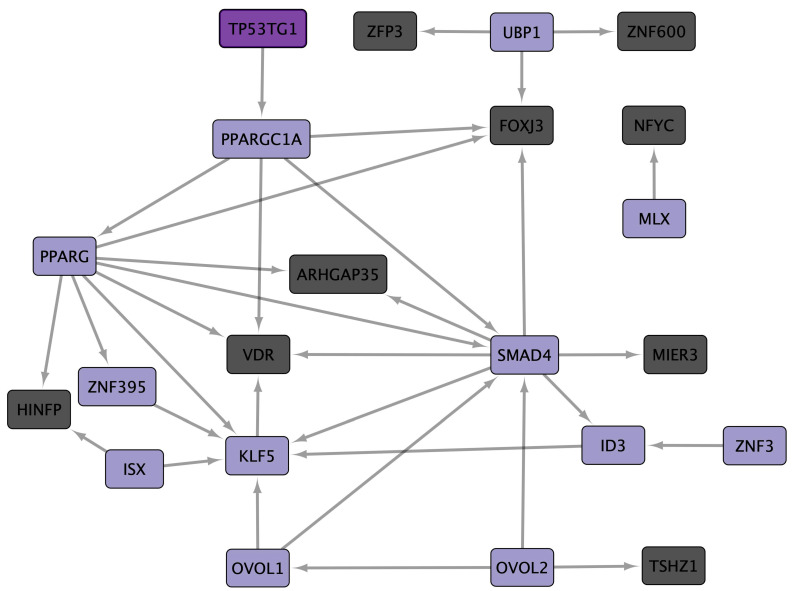
Coding (lilium and grey) and non-coding (purple) transcriptional regulatory network (cncTRN) of key upregulated transcription factors in CD. Created with Cytoscape.

Zinc finger protein 91 (ZNF91) is common in ten CD datasets, eight TFs are common in nine CD datasets, and the lncRNA TP53 target 1 (TP53TG1) and the other twenty-two TFs are common in eight CD datasets. cncTRN analysis identified TP53TG1 and another twelve TFs as key regulators in CD (Figure 3). ZNF91 is also a key regulator according to CD GRN, along with another 29 TFs, and regulates 374 CD common overregulated DEGs, including TP53TG1, which are co-expressed in CD, colon cancer, and lung adenocarcinoma and are related to transmembrane transport, mitochondrial function, and multiple metabolic pathways and diseases (Table S1.6). Annotation analysis identified CD TFs related to positive and negative regulation of transcription and form transcription regulator complexes. CD upregulated DEGs are related to the function of transit peptides, which are responsible for the transport of a protein encoded by a nuclear gene to a particular organelle, as well as the mitochondria, membrane, peroxisome, endoplasmic reticulum, endosome, extracellular exosome, golgi stack, and receptor complex. Upregulated DEGs are involved in several metabolic signaling pathways, like fatty acid degradation, along with PPAR (PPARG and PARGC1A), MAPK (VDR), SUMOylation of intracellular receptors (PPARG, THRB, and VDR), nuclear receptor transcription (PPARG, THRB, and VDR), Erb, and peroxisome.

Upregulated DEGs and TFs are also involved in several cellular and biological processes that might be related to the acquisition of the hallmarks of cancer, like metabolism, cell differentiation, cell proliferation, angiogenesis, apoptosis, cell development, cell migration, response to growth factor, response to cytokine, response to hormone, and epigenetics reprogramming (Table 3). CD has thirty-four overregulated DEGs, including membrane receptors and TFs previously involved in cancer. According to our previous transcriptomic analysis [36], CD and lung cancer have nine (ALDH18A1, DSP, GNG4, LAD1, PFN2, PPP2R3A, PTPRF, and SFXN1) common overregulated genes located in the mitochondrion, extracellular exosome, membrane, and receptor complex related to metabolic pathways, ATP binding, epigenetic reprograming through dephosphorylation, and acetylation. CD has no common overregulated genes with gastric cancer and one gene (TMTC4) and one TF (ZNF3) with colon cancer, located in the endoplasmic reticulum and membrane.

### 3.3. Ulcerative Colitis (UC)

**Figure 4:**
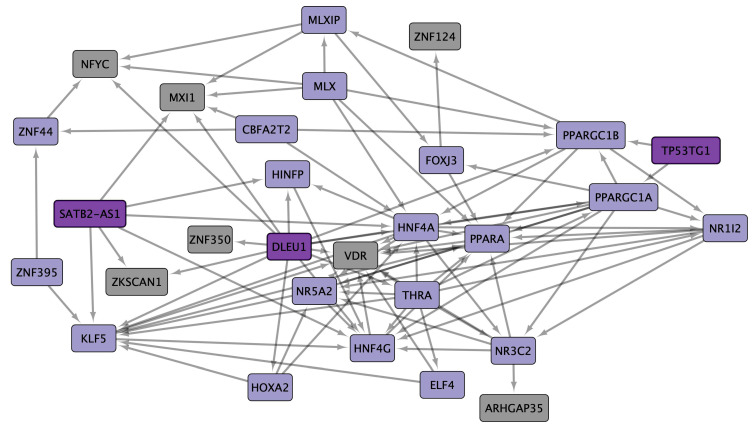
Coding (lilium and grey) and non-coding (purple) transcriptional regulatory network (cncTRN) of key upregulated transcription factors in ulcerative colitis. Created with Cytoscape.

ZNF91 and vitamin D receptor (VDR) are common in eleven UC datasets; thirteen TFs are common in ten UC datasets; thirty-three TFs and the three lncRNA (Deleted in lymphocytic leukemia 1 (DLEU1), Special AT-rich sequence binding protein 2 antisense RNA1 (SATB2-AS1), and TP53TG1) are common in nine UC datasets. cncTRN analysis identified DLEU1, SATB2-AS1, TP53TG1, and nineteen other TFs as key regulators in UC (Figure 4). VDR is also a key regulator according to UC GRN, along with 44 other TFs, and regulates 409 UC common overregulated DEGs, including TP53TG1 and SATB2-AS1, which are co-expressed in colon cancer and related to transmembrane transporter activity, phosphorylation, mitochondrial and peroxisome function, PPAR, and multiple metabolic pathways and diseases. ZNF91 is another key regulator according to UC GRN, along with 44 other TFs, and regulates 397 UC common overregulated DEGs, co-expressed in colon cancer and related to transmembrane transport, mitochondrial function, and multiple metabolic pathways and diseases (Table S1.9). Annotation analysis identified UC TFs related to positive and negative regulation of transcription, and form transcription regulator complexes related to transcription coregulator binding. UC upregulated DEGs are related to the function of mitochondria (MLXIP and MTERF2), transit peptides (MTERF2), extracellular exosomes, nuclear receptor activity (HNF4A, HNF4G, NR1H4, NR1I2, NR3C2, NR5A2, PIK3R1, PPARA, THRA, and VDR), membranes (BZW2 and ZNF219), peroxisome, endoplasmic reticulum, endosome, and membrane raft. Upregulated DEGs are involved in numerous metabolic signaling pathways, like fatty acid degradation, along with AMPK (PPARGC1A and HNF4A), PPAR (PPARA), SUMOylation of intracellular receptors, nuclear receptors transcription (HNF4A, HNF4G, NR1H4, NR1I2, NR3C2, NR5A2, PPARA, THRA, and VDR), calcium, and peroxisome.

Upregulated DEGs and TFs are also involved in several cellular and biological processes that might be related to the acquisition of the hallmarks of cancer, like the regulation of metabolic processes, circadian rhythm, cell differentiation, cell proliferation, cell development, response to lipids, response to hormones, and epigenetics reprogramming (Table 4). According to our previous transcriptomic analysis [36], UC and lung cancer have seven (ALDH18A1, CASK, CNTNAP2, DNAJA3, MDH2, PPP2R3A, and PTPRF) common overregulated genes and one TF (BZW2) located in mitochondrion, membrane, and extracellular exosome, and related to metabolic pathways, citrate cycles, carbon, pyruvate, glyoxylate and dicarboxylate metabolism, ATP binding, transit peptides, and epigenetic reprograming through acetylation. UC has no common overregulated genes with gastric cancer or colon cancer.

## 4. Discussion

The transcriptomic analysis of PAH, CD, and UC identified two common overregulated DEGs between the three inflammatory diseases (Figure 1). PRUNE1 is a short-chain phosphatase of the aspartic acid-histidine-histidine family, with multiple transcript splicing variants, and with nucleotide exopolyphosphatase/phosphodiesterase function, in-cell migration, and proliferation [43]. PRUNE1 has not been previously analyzed in gut and lung inflammatory diseases, but stimulates tumorigenesis, progression, and metastases through the induction of lung and gut cell motility [44]. TSPAN7 is a cell surface glycoprotein of the transmembrane 4 superfamily, characterized by lateral organization with other membrane proteins to form tetraspanin-enriched microdomains that influence cell adhesion, migration, invasion, and survival, and which induce downstream signal transduction [45]. TSPAN7 has not been previously analyzed in gut and lung inflammatory diseases but promotes the migration and proliferation of lung cancer cells via epithelial-to-mesenchymal transition [46], regulates several types of cancer cell growth, metastasis, stemness, drug resistance, and biogenesis of extracellular vesicles (exosomes and migrasomes), and the immune-microenvironment [45]. PAH, CD, and UC common genes are involved in epigenetic reprograming and metabolic pathways (Table S1.10). PAH and CD common genes are related to the function of nucleoplasms and extracellular exosomes. PAH and UC common genes are related to the golgi, endoplasmic reticulum, extracellular exosome, mitochondrion, and nucleoplasm function. CD and UC common genes are involved in mitochondrion and extracellular exosome function, multiple metabolic pathways, epigenetic reprograming, PPAR and AMPK signaling pathways, as well as proteoglycans in cancer. Therefore, PAH, CD, and UC common overregulated genes suggest the importance of cell–cell communication, epigenetic reprograming, metabolic pathways, and the capacity to acquire the hallmarks of cancer during lung and gut cell adaptation to inflammatory phenotypes.

### 4.1. Coding and Non-Coding Transcriptional Regulators of Gut and Lung Inflammatory Diseases

**Figure 5:**
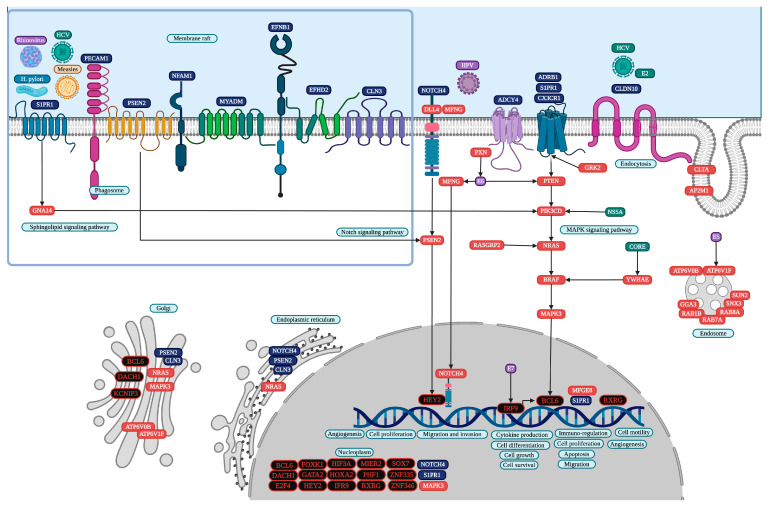
Microbiome interaction with membrane receptor of PAH-related cells activating signaling pathways involved in transcriptional regulation during lung inflammation. In red are the upregulated genes and TFs; in black are the key upregulated TFs. Created with BioRender.com.

PAH is a disease characterized by elevated mean pulmonary artery pressure, increased vascular resistance, and remodeling of the pulmonary vascular bed, which ultimately leads to right heart failure and death [47]. PAH patients have characteristic inflammatory responses, and immunomodulatory interventions have been able to control disease development and progression, while inflammation induces the entry of innate and adaptive immune cells into the pulmonary vascular wall, increasing cytokine and chemokine levels in the blood and perivascular tissue of pulmonary arteries [48]. Five PAH datasets were analyzed [49,50,51,52,53,54,55,56], showing a statistically significant overexpression of 24 common TFs in at least three datasets of our transcriptomic analysis (Table S1.1). Moreover, PAH cncTRN analysis identified IRF9, LINC01089, and other TFs as key transcriptional regulators of this inflammatory disease, capable of regulating the other TFs (Figure 2). IRF9 is essential for the transcription of interferon-stimulated genes involved in the Type I and III interferon (IFN) signaling pathways, which are critical for innate immune responses to viral infections and other stress signals; then, its dysregulation can lead to excessive inflammation with the production of pro-inflammatory cytokines (e.g., IL-6 and TNF-α), which overrides the benefits of the IFN-III response on intestinal epithelial cells, contributing to chronic inflammatory diseases and creating a tumor-promoting environment [57]. IRF9 accelerates the characteristic abnormal proliferation in pulmonary artery smooth muscle cells in PAH by regulating prohibitin 1 (PHB1) expression and leading to mitochondrial dysfunction, including genes linked to endosomes and lysosomal enzymes [58], suggesting that targeting IRF9 may be a novel strategy to delay PAH pathological progression [59], perhaps also through the regulation of HPV E7, which might directly regulate IRF9 expression (Figure 5).

Signaling pathways mediated by IRF9 might contribute to chronic inflammation under pathological conditions, increasing reactive oxygen species and DNA damage, angiogenesis, cell proliferation, and resistance to apoptosis, particularly when the regulatory balance of IFN signaling is disrupted, increasing the likelihood of mutations that drive tumorigenesis in the gut [58]. IRF9 signaling has been implicated in the epithelial–mesenchymal transition, a hallmark of cancer metastasis [60], suggesting that it could facilitate cancer progression. IRF9 is involved in immunomodulation, cell cycle regulation, cell survival, and cell differentiation, impairing immune surveillance, which allows tumor cells to evade immune detection [61]. The key role of IRF9 in modulating immune responses may influence the efficacy of immune checkpoint therapies [62], as the impact on interferon-stimulated gene expression might affect tumor resistance to immune-based treatments. IRF9-mediated signaling might influence microbial-driven inflammation and tumorigenic processes. In normal conditions, IRF9 might enhance the antimicrobial and inflammatory functions of macrophages and dendritic cells by promoting the expression of genes involved in pathogen recognition and destruction, increase neutrophil recruitment and activation by upregulating chemokines, and support NK cell activation and the production of IFN-γ, amplifying microbial-driven inflammation [58]. Therefore, it is crucial to identify how IRF9 interacts with other coding and non-coding transcriptional regulators throughout signaling pathways in the tumor microenvironment to control the expression specifically of inflammation- and cancer-related interferon-stimulated genes and to develop advanced therapeutic strategies against these pathological processes. LINC01089 is a super enhancer-driven lncRNA that induces epithelial–mesenchymal transition, migration, invasion, and metastasis by regulating DIAPH3 alternative splicing, blocking N6-methyladenosine-mediated mRNA stabilization, and establishing an epigenetic network that promotes hepatocellular carcinoma metastasis [63]. LINC01089 has shown tumor-suppressive effects in several cancer types [64]. According to PAH GRN analysis, IRF9 is capable of regulating LINC01089 expression along with 189 other PAH common DEGs, which are known to be co-expressed in lung cancer models and are related to retromer complex binding. The highly conserved retromer pathway might be an important target of intracellular viruses and intravacuolar bacteria, involved in mechanisms to modulate host endosomal trafficking, suggesting the importance of the IRF9 regulatory function in the control of intracellular microbial growth mediated by bacterial effectors, such as chlamydia trachomatis IncE and legionella pneumophila RidL [65]. Then, IRF9 is a known regulator of PAH chronic inflammation and innate immune responses to viral infections, while LINC01089 has not been previously analyzed within PAH pathogenesis, though both are modulators of tumorigenic processes, suggesting their importance in PAH inflammatory phenotypes and the early stages of cancer.

**Figure 6:**
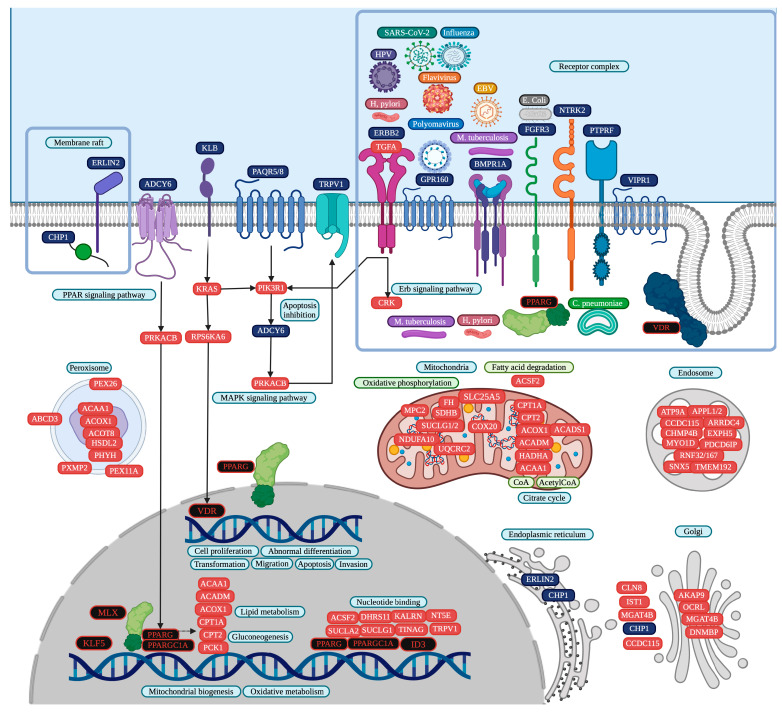
Microbiome interaction with membrane receptor of CD-related cells, activating signaling pathways involved in transcriptional regulation during gut inflammation. In red are the upregulated genes and TFs; in black are the key upregulated TFs. Created with BioRender.com.

**Figure 7:**
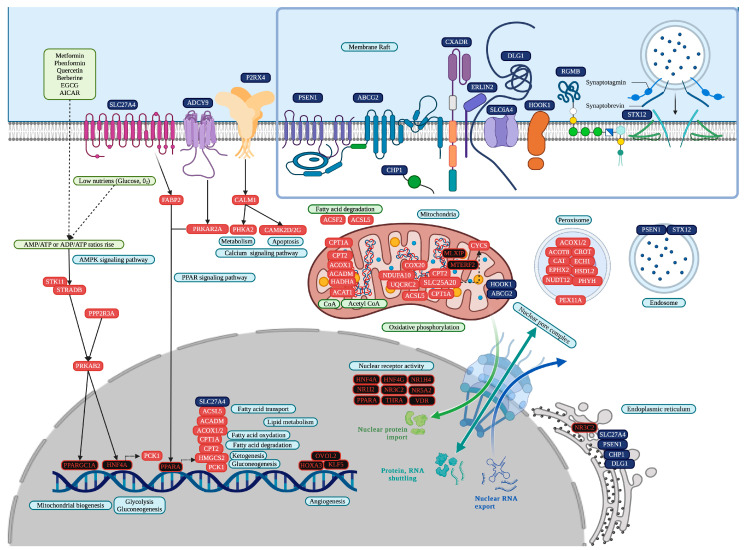
Microbiome interaction with membrane receptor of UC-related cells, activating signaling pathways involved in transcriptional regulation during gut inflammation. In red are the upregulated genes and TFs; in black are the key upregulated TFs. Created with BioRender.com.

IBDs are chronic diseases of the gastrointestinal tract mucosa with a multifactorial and still not fully known etiopathogenesis, characterized by the presentation of two main types. CD causes inflammation anywhere in the gastrointestinal tract, from the mouth to the anus, while UC causes inflammation and ulceration in the large intestine, compromising the colon and rectum. Moreover, the prevalence of CD and UC is higher in younger individuals, disrupting half of their life, and it is related to geographical location (as both are more often diagnosed in urban areas), genetics, inappropriate diet, and immune response [66]. Twelve CD datasets were analyzed [67,68,69,70,71,72,73,74,75,76,77,78], showing a statistically significant overexpression of 31 common TFs in at least eight datasets (Table S1.4). Moreover, CD cncTRN analysis identified TP53TG1 and other TFs as key regulators of this inflammatory disease, capable of regulating the other TFs (Figure 3), which might be related to the regulation of several metabolic, PPAR, MAPK, Erb, and peroxisome signaling pathways (Figure 6). Twelve UC datasets [67,68,70,71,73,78,79,80,81,82,83,84] were analyzed, showing a statistically significant overexpression of 48 common TFs in at least nine datasets (Table S1.7). Moreover, UC cncTRN analysis identified DLEU1, SATB2-AS1, TP53TG1, and other TFs as key regulators of this inflammatory disease (Figure 4), which might be related to the regulation of numerous metabolic, AMPK, PPAR, calcium, and peroxisome signaling pathways (Figure 7). ZNF91 is a TF of the Krüppel-associated box subfamily required to repress SINE-VNTR-Alu (SVA) retrotransposons, which recognizes and binds SVA sequences and represses their expression by recruiting a repressive complex containing TRIM28/KAP1 [85]. ZNF91 is related to poor prognoses of esophageal cancer patients, to AML cell proliferation and anti-apoptosis, to irradiation resistance by regulating the stem cell-like properties of NSCLC cells, and to the occurrence and development of colorectal cancer [86]. The ZNF91 subfamily has continued to expand and diversify throughout the evolution of apes and humans, suggesting a role in determining gene expression differences in humans and the evolution of novel traits, through repressive DNA methylation and H3K9me3 at repeats along the human genome [87]. TP53TG1 was discovered as a target gene of TP53, related to DNA methylation-associated silencing that produces aggressive tumors resistant to cellular death when DNA-damaging agents and small targeted molecules are used, and to a role in the p53 response to DNA damage [88]. The knockdown of TP53 in cancer cells causes the upregulation of TP53TG1, positively regulating cell proliferation and migration, but reducing intrinsic ERK signaling [89]. According to CD GRN analysis, ZNF91 is capable of regulating the expression of TP53TG1 along with 374 CD common overregulated DEGs, which are known to be co-expressed in CD, colon cancer, and lung adenocarcinoma, related to transmembrane transport, mitochondrial function, and multiple metabolic pathways and diseases. In UC GRN analysis, ZNF91 regulates 397 UC common overregulated DEGs, which are known to be co-expressed in colon cancer, related to transmembrane transport, mitochondrial function, and multiple metabolic pathways and diseases. ZNF91 is overregulated in ten CD datasets and eleven UC datasets, while TP53TG1 is overregulated in eight CD datasets and nine UC datasets, suggesting that they might be key regulators of IBD gene expression through transcriptomic and epigenomic mechanisms involved in mitochondrial function and metabolic pathways; however, their function has not been analyzed yet in these inflammatory diseases, neither in its association with microbiome network formation nor its function.

VDR is a member of the nuclear hormone receptor superfamily of ligand-inducible TFs, and its downstream targets are principally involved in several metabolic pathways, such as those involved in immune response and cancer [90]. VDR is expressed in the colonic epithelium and correlates with UC severity and inflammation status [91]. Nuclear receptors may play critical roles in inflammatory- and metabolic-related diseases (Figure 7). The association of VDR and microbial metabolites has already been established, as it is involved in immunomodulation, proliferation, and autophagy through specific effector molecules, which directly influences innate immunity and epigenetic modulation of the host [92,93]. VDR plays a critical role in regulating gut microbiota through its influence on the immune system, influencing the production of cytokines, epithelial barrier integrity controlling the expression of tight junction proteins, and microbial composition controlling the expression of genes involved in bile acid pathways producing signaling molecules and antimicrobial agents, and the balance of pathogenic bacteria (e.g., Helicobacter species) and beneficial bacteria (e.g., Lactobacillus and Bifidobacterium) [92]. VDR interacts with various molecular partners, including other receptors, TFs, and signaling molecules. VDR interacts with PPAR in pathways regulating cell proliferation and differentiation inflammation, metabolism, and adipogenesis, affecting processes like insulin sensitivity and immune modulation [94].

DLEU1 is an lncRNA identified as a potential tumor suppressor gene downregulated in B-cell chronic lymphocytic leukemia patients, with over twenty different splice variants involved in tumorigenesis of several types of cancer, including non-small cell lung cancer, colorectal cancer, and gastric cancer [95]. DLEU1 upregulation increases cell viability, migration, invasion, decreased apoptosis, and the epigenetic regulation of TFs during tumorigenesis, for example, through the interaction with miRNAs and by inducing histone modifications and phosphorylation across mTOR signaling [96]. DLEU1 is related to VDR regulation (Figure 7), suggesting an association with microbial function during UC inflammatory processes. SATB2 is a nuclear matrix-associated protein member of the SATB family of proteins that serves as a key regulator of high-order chromatin organization, and the expression of miR-31 promotes tumorigenesis in UC-associated neoplasia via downregulation of SATB2 [97]. SATB2-AS1 is an lncRNA related to tumor growth, lung metastasis, and the tumor immune microenvironment by regulating SATB2, and both might be biomarkers for risk stratification and therapeutic targeting [98]. SATB2-AS1 inhibits tumor metastasis, affects the tumor immune cell microenvironment in colorectal cancer by regulating SATB2 [99], and mediates epigenetic regulation of SATB2 and Snail expression to suppress colorectal cancer progression and aggressiveness by inhibiting SATB2-dependent snail transcription and epithelial–mesenchymal transition [100]. The coding and non-coding RNAs identified in the cncTRN analyses demonstrate the diverse regulatory interactions between lncRNAs and mRNA, highlighting their significant roles in inflammation, cancer establishment, and progression, as well as future potential therapeutic targets against gut and lung inflammatory and tumorigenic diseases [101]. According to UC GRN analysis, VDR is capable of regulating the expression of TP53TG1 and SATB2-AS1 along with 409 CD common overregulated DEGs, which are known to be co-expressed in colon cancer, and related to transmembrane transporter activity, phosphorylation, mitochondrial and peroxisome function, PPAR, and multiple metabolic pathways and diseases. Then, VDR and DLEU1 are known regulators of gene expression in UC, involved in inflammatory, immune, metabolic, and tumorigenic pathways. SATB2 is a TF known to be deregulated in UC; however, the function of its SATB2-AS1 has only been related to tumor growth and the microenvironment. Only VDR has an established association with microbiome network formation and function, while DLEU1 association might be indirect through VDR regulation.

### 4.2. Host–Microbiota Crosstalk Through Lung and Gut Cells Receptors Present in Membrane Rafts and Complexes

Lung, gastric, and colon cancer cells interact with several microorganisms [4]. In our PAH transcriptomic analysis, HPV and HCV infection might be important in PAH pathogenesis, which may interact with specific membrane receptors or viral proteins (HPV-E6, HCV-NS5A, and HCV-CORE) to activate MAPK and NOTCH signaling pathways, and in turn to regulate TF expression like IRF9, BLC6, HEY2, and RXRG (Figure 5). Several microorganisms have been implicated as IBD etiologic agents, like mycobacterium tuberculosis, diplostreptococci, bacteroides fragilis, bacteroides necrophorum, helicobacter hepaticus, helicobacter pylori, listeria, pseudomonas maltophilia, pathogenic escherichia coli, chlamydia, shigella, coxsakie A and B, wolinella, Norwalk virus, polio virus, reovirus, herpes virus, paramyxovirus, and influenza B [102]. However, there is still no direct evidence involving the common IBD upregulated DEGs with signaling pathways of microbial infections; therefore, the receptors in membrane rafts and complexes were analyzed to infer some host–microbiota interactions with the regulation of signaling pathways. Membrane rafts are small dynamic domains of 10–200 nm with cholesterol and sphingolipids for specific cellular processes, essential to assembly and budding events, as well as virus interaction with the cell surface and virus entry to the host cells through endocytic and non-endocytic mechanisms [103]. Receptor complexes are specific arrangements of signaling proteins that respond to extracellular signals, facilitating communication between the extracellular environment and the intracellular signaling pathways, leading to a conformational change that activates intracellular signaling cascades, thus targeting key protein–protein interactions within receptor complexes and providing an opportunity to develop more selective drugs with fewer side effects [104]. PAH and UC have overregulated genes related to membrane rafts, while CD has overregulated genes related to a receptor complex.

The PAH membrane raft has eight membrane receptors (CLN3, EFHD2, EFNB1, MYADM, NFAM1, PECAM1, PSEN2, and S1PR1), which might be related to extracellular vesicles and microorganisms’ entry into host cells (Figure 5) and that are mostly regulated by IRF9 (Table S1.3). Sphingosine-1-phosphate receptor 1 (S1PR1) is a G-protein-coupled receptor that has an important role in regulating critical cell functions, including cell growth, apoptosis, differentiation, migration, and activation through the sphingolipid metabolic pathway, with several of its downstream effectors localized in membrane microdomains named lipid rafts [105]. S1PR1 plays a role in the maturation, activation and chemotaxis of immune cells, specifically mediating migration and differentiation of macrophages, and presents a potential antigen for PAH autoimmunity, given the documented role of S1P/S1PR signaling in PAH pathogenesis [106]. S1PR1 may play important roles in cancer progression in the context of chronic inflammation [107]. S1PR1 inhibits apoptosis, activating MAPK signaling and reducing ROS levels in AML cells and inducing proliferation in HCC and esophageal squamous cell carcinoma [108]. The MAPK signaling pathway might function as a mediator of cellular stresses, such as inflammation and apoptosis during PAH and tumorigenic processes, regulating the expression of key TFs (Figure 5), while sphingolipid metabolites play a critical role in the host’s defense against intracellular pathogens, as helicobacter pylori and viruses such as HCV, rhinovirus, and measles, which are dependent on sphingolipids present in the host cell membrane, attempt to gain cellular entry [109]. S1PR1 can also interact with various immune cells, including lymphocytes, to modulate immune responses, with endothelial cells to regulate blood vessel formation and response to inflammatory signals, with components of the extracellular matrix influencing cellular adhesion and migration [110] to activate signaling pathways like MAPK and induce cell proliferation and differentiation (Figure 5). EFHD2, EFNB1, MYADM, NFAM1, and PECAM1 may also be related to MAPK signaling pathways, leading to inflammation and recruitment of immune cells, while PSEN2 might be related to NOTCH signaling, as presenilins are involved in the cleavage of NOTCH receptors [111]. There is not yet research that has identified the specific microorganisms that might interact with these receptors, but their presence in the PAH membrane raft and its role in inflammatory and immune regulation suggests an important function in cell–cell communication and host–pathogen interactions during PAH, lung, and gut tumorigenesis.

Membrane rafts and receptor complexes play a significant role in the activation and regulation of the PPAR signaling pathway, during which PPAR proteins that combine TF and receptor molecule functions are regulated by fatty acid signals derived from dietary lipids, pathogenic lipoproteins, or essential fatty acid metabolites to control lipid metabolism differentiation, metabolism, immunity, and inflammation, and thus have the unique ability to bind lipid signaling molecules and transduce the appropriate signals derived from lipid environments to the level of gene expression [112]. PPAR D domain links the DNA binding domain to the C-terminal, acting as a docking site for co-factors; the N terminal or E/F domain recruits co-factors to assist the transcription process via the ligand-dependent transactivation function, and the amino-terminal A/B domain is poorly conserved between the three isotypes, which contain a ligand-independent activation function-1 [113]. AMPK and MAPK phosphorylate the amino-terminal A/B domain to modulate PPAR receptor activity in an isotype-dependent manner, regulating their transcriptional activity, which is involved in metabolism and inflammation, inducing tumorigenic processes [114]. CD and UC overregulated DEGs, including some TFs (MLX, MLXIP, PPARA, PPARGC1A, and PPARGC1B), are related to insulin resistance, as insulin can stimulate PPARα-mediated transcription via MAPK-induced phosphorylation at Ser12 and Ser21, adjusting PPAR activity to match cellular demands for growth, differentiation, and survival [113]. PPARs also influence fatty acid degradation by promoting β-oxidation and preventing the buildup of lipid intermediates, while fatty acid degradation is the process in which fatty acids are broken down into their metabolites by beta-oxidation in mitochondria and peroxisomes and by omega-oxidation in microsomes, generating acetyl-CoA, the entry molecule for the citric acid cycle, the main energy supply of cells, including bacteria and cancer cells, where PPARs promote their own survival and progression, altering metabolic patterns and regulating lipid metabolism [115].

CD has a receptor complex of nine membrane receptors (BMPR1A, ERBB2, FGFR3, GPR160, NTRK2, PPARG, PTPRF, VIPR1, and VDR) (Figure 6), and they are mostly regulated by ZNF91 (Table S1.6). Polyomaviruses, flaviviruses, influenza virus, and SARS-CoV-2 activate and/or require GPCR-mediated pathways for cell infection, including MAPK [116], suggesting that GPR160 could become an attractive target for the development of broad CD antiviral therapies. Mycobacterium tuberculosis might influence BMP signaling, modifying immune responses in CD through BMPR1A-specific interactions, where the inflammatory host milieu might also affect expression of mycobacterial factors such as DosR and WhiB3 to co-ordinate pathogenic evasion [117], activating the MAPK non-canonical signaling pathway [118]. Epstein–Barr virus (EBV) interacts with TGF-β signaling, which is closely related to BMP, to suppress immune responses; therefore, it might also influence BMP receptor pathways indirectly [119]. Helicobacter pylori activates ERBB2 through the production of cytotoxins, which can lead to aberrant signaling, inflammation, and increasing gastric cancer risk, forming homo-dimers or hetero-dimers, which activate phosphorylation cascades within the cell, in turn leading to activation of Erb and MAPK signaling pathways, driving proliferation and survival of cancer cells [120]. HPV can upregulate ERBB family receptors, enhancing proliferation and survival of cancer cells, and HPV E6/E7 might cooperate with ErbB-2/HER2 receptors to trigger cellular transformation through D-type cyclins in CD [121]. Certain strains of Escherichia coli have been shown to influence FGF signaling; FGFR3 activation by FGFs stimulates RAS, which then activates the MAPK pathway to promote cell survival, growth, and differentiation during infection [122]. Chlamydia pneumoniae is respiratory pathogen able to activate PPARG in macrophages through the upregulation of acetyl-CoA acetyltransferase 1 (ACAT1) [123], so it might upregulate another acetyl-CoA to suppress inflammatory responses, aiding in bacterial survival of CD cells. The PPARG N-terminus A/B domain is phosphorylated by MAPK, which modifies its nuclear receptor activity, influencing cell differentiation and inflammatory responses [124]. Helicobacter pylori can activate PPARG in gastric cells, controlling MMP-10 expression through the MAPK signaling pathway and triggering its downstream target catalase, which helps reduce the immune response, creating a more favorable environment for bacterial survival [125]. Mycobacterium tuberculosis and its cell wall mannose-capped lipoarabinomannan induce PPARG expression through a macrophage mannose receptor-dependent pathway, which promotes IL-8 and cyclooxygenase 2 expression, and upstream MAPK-p38 mediates cytosolic phospholipase A2 activation, required for PPARG ligand production [126]. Transcription regulation by steroid hormones and other metabolites is mediated by nuclear receptors such as the vitamin D and retinoid X receptors (VDR and RXR), as VDR binds to its ligand, vitamin D, and forms a complex with RXR (Retinoid X Receptor) that regulates genes involved in calcium and phosphate homeostasis, immune response, and cellular differentiation [127]. PPARG is the central player in PPAR signaling, which lastly regulates genes involved in lipid uptake, glucose metabolism, adipocyte differentiation, and inflammatory responses [128]. VDR is expressed by macrophages, and CYP27B1 is a critical enzyme in the vitamin D metabolic pathway and, in CD, CYP2B6 may be critical for the metabolic signaling pathways regulated that regulate target genes known to be induced by the VDR-complex, acting in an intracrine mechanism by interacting with VDR to induce the expression of innate immune responses in macrophages to control bacterial challenges like mycobacterium tuberculosis [129]. VDR may not interact directly with microorganisms, but the association of VDR and microbial metabolites has been already stablished, and its activation plays a significant role in the host’s ability to respond to and control infections by regulating immune responses and enhancing the expression of antimicrobial peptides [130]. The CD receptor complex is composed of membrane receptors that might be activated by a characteristic microbiome inflammatory network, related to the regulation of PPAR and MAPK signaling pathways, and the control of gene expression of key metabolic processes during the progress of this inflammatory disease.

UC has a membrane raft of ten membrane receptors (ABCG2, CHP1, CXADR, DLG1, ERLIN2, HOOK1, PSEN1, RGMB, SLC6A4, and STX12) (Figure 7), two of them (CHP1 and ERLIN2) in common with CD (Figure 6), and which are mostly regulated by VDR and ZNF91 (Table S1.9). ABCG2 is an efflux transporter often found in lipid rafts of gut epithelial cells that may protect the mucosal barrier by transporting the inflammatory metabolites and xenobiotics of cells, which may be involved in the cellular homeostasis of cholesterol, as well as in the regulation of its content in lipid rafts, regulating the function of membrane proteins, including receptors of the innate immune system, which may also regulate lipid transport and participate in phagocytosis, inflammation, and perform other functions of innate immunity [131]. Bone-marrow mesenchymal stem cells act as a useful niche for mycobacterium tuberculosis, wherein they can survive by escaping the antibiotic assault that can be attributed to the host ABCG2 efflux pumps; then, their inhibition in UC can become an adjunctive chemotherapy to eliminate the bacteria from this protective niche [132]. PPARG directly regulates the transcription of ABCG2; therefore, its upregulation can be achieved via exogenous or endogenous activation of the lipid-activated TF PPARG, which can significantly modify the xenobiotics and drug resistance of human myeloid dendritic cells [133]. CXADR Ig-like cell adhesion molecules or junctional adhesion molecules (JAMs) are a type I membrane receptor for group B coxsackieviruses and subgroup C adenoviruses that increase the barrier function in cultured epithelial cells, suggesting a functional role in tight junction formation or maintenance [134]. ERLIN2 is an endoplasmic reticulum membrane lipid raft with homologous proteins in caenorhabditis elegans and arabidopsis thaliana that participates in the degradation of inositol 1,4,5-triphosphate receptors, and in detergent-resistant membranes forms high molecular weight complexes containing homo- and hetero-oligomers [135]. Under UC, endoplasmic reticulum stress could be important for efficient hepatitis C virus infection as it influences ERLIN2’s role in lipid raft stability, perhaps regulating the expression of PPARGC1A and PPARGC1B [136], having an impact in lipid metabolism and homeostasis, a process controlled in part by the PPAR signaling pathway [137]. Calcineurin-like EF-hand protein 1 (CHP1) is involved in maintaining intracellular pH and ion balance by regulating ion exchangers and homeostasis, which is crucial for cell signaling, especially within lipid rafts; therefore, disturbances in CHP1 function disrupt ion transport in UC and CD epithelial cells, leading to dysregulated signaling and contributing to inflammation through impaired epithelial barrier function and cellular stress [138]. Discs Large MAGUK Scaffold Protein 1 (DLG1) is a multi-domain scaffolding protein with multiple alternative splicing variants key in septate junction formation, signal transduction, cell proliferation, and lymphocyte activation; therefore, its dysfunction in UC may lead to impaired cell–cell adhesion, compromising the epithelial barrier, tight junction integrity in lipid rafts, and contributing to increased gut permeability and inflammatory signaling [139]. DLG1 is targeted by HPV [140] and the avian influenza virus NS1 ESEV PDZ binding motif [141], which may lead to disrupted epithelial cell junctions and enhanced viral replication, influencing infection in gastrointestinal mucosa in UC, while HPV-18 E7 is also able to increase DLG1 levels, likely by rescuing it from E6-mediated proteasomal degradation [142]. DLG1 may recruit components of the vesicle trafficking machinery either to the plasma membrane or to transport vesicles, participating in vesicle formation, targeting, tethering, and fusion, in exocytotic and endocytotic pathways that contribute to membrane homeostasis and insulin stimulation of glucose transport [143]. Hook microtubule tethering protein 1 (HOOK1) is a coiled-coil protein with several alternatively spliced transcript variants, which bind to microtubules and organelles through their N− and C-terminal domains, respectively, and which interacts with several members of the Rab GTPase family involved in intracellular transport, including endosomal trafficking, and which may influence membrane raft composition by transporting key lipid raft components [144]. Therefore, HOOK1-related trafficking defects could disrupt the delivery of proteins or lipids needed to maintain raft stability and organization, affecting immune cell signaling and contributing to inflammation in UC. Chlamydia trachomatis is a vacuole-bound pathogen that manipulates host-cellular functions to invade host cells and maintain a replicative niche, participating in endosomal trafficking and intracellular transport mechanisms to enhance its survival [145], where it might interact with HOOK1 to prevent lysosomal fusion and degradation to acquire energy and nutrients essential for its survival and replication [146]. Presenilin 1 (PSEN1) is a part of the gamma secretase complex involved in cleavage of transmembrane proteins, and in lipid rafts it might be able to regulate immune and inflammatory signaling pathways and gut epithelium cell renewal [147]. Repulsive Guidance Molecule B (RGMB) is a glycosylphosphatidylinositol-anchored member of the RGM family related to embryonic development, tumorigenesis, and immune and intestinal epithelial cell interactions with the gut lining [148]; its deficiency induces a predominant gut microbiota imbalance under basal and inflammatory conditions as it mediates prevotellaceae loss [149]. Microbiota dysbiosis causes the upregulation of PD-L2 and its receptor RGMB on CD8+ T cells, thus explaining the resistance of cancers to PD-L1 blockades [150]. SLC6A4 is the transporter of the neurotransmitter serotonin through synaptic spaces; in UC, it might regulate serotonin reuptake, impacting gut motility, immune function, and pro-inflammatory signaling, worsening symptoms [151]. Bacillus subtilis, enterococcus faecium, and enterococcus faecalis can upregulate the expression level of the serotonin transporter in intestinal cells in a concentration-dependent manner, and could be assessed by possible substances, such as bacterial components or small-molecule proteins regulating the serotonin transporter expression level [152]. Syntaxin 12 (STX12) enables SNAP receptor activity and SNARE binding activity involved in the ordered fusion of endosomes and lysosomes with the phagosome when phagocytic cells kill and degrade internalized foreign particles. Protein stabilization, vesicle fusion [153], including immune receptor endocytosis and transporter recycling crucial for membrane composition, potentially amplify inflammation [154]. The UC membrane raft is composed of membrane receptors that might be activated by a characteristic microbiome inflammatory network, related to the regulation of PPAR and AMPK signaling pathways, and the control of gene expression of key metabolic processes during this inflammatory disease.

### 4.3. Transcriptomic Association Between Gut and Lung Inflammatory and Tumorigenic Diseases

The analysis of the possible association between gut and lung inflammatory and tumorigenic diseases identified common overregulated genes. PAH and lung cancer association through physio-pathogenic mechanisms has previously been suggested in several studies [10], with evidence that shares processes characteristic of lung cancer that point to a possible causal association in the acquisition of the hallmarks of cancer [20]. According to our transcriptomic analysis, PAH has common overregulated genes with lung, gastric, and colon cancer (Figure 1), located in the nucleoplasm, transmembrane helix, endoplasmic reticulum membrane, lysosomal membrane, and extracellular exosomes, and related to regulation of transcription and gene expression epigenetic reprograming, apoptosis, proliferation, and symbiont entry into the host cell, suggesting their importance in intracellular transport, cell–cell communication, and the epigenetic regulation of gene expression related to the acquisition of the hallmarks of cancer (Table S1.10). AEBP1 is the AE binding protein 1, a transcriptional regulator common in PAH and colon cancer that positively controls MAP-kinase activity, a key signaling pathway identified in the annotation analysis (Figure 5), leading to enhanced cell proliferation and reduced cell differentiation, inflammation, macrophage cholesterol homeostasis, and atherogenesis [155]. AEBP1 overexpression promotes epithelial–mesenchymal transition [156]. ZNF503 is a transcriptional regulator of the zinc finger protein family, common in PAH and colon cancer, involved in G1 to G0 transition during cell differentiation, development, and tumor initiation, enhancing the proliferation of cancer cells, and inhibiting the expression of other TFs like GATA3, which is related to poor prognostics, an advanced TNM stage, venous invasion, migration, and epithelial–mesenchymal transition progress [157]. AEBP1 and ZNF503 have not been previously related with PAH pathogenesis, and both were identified as common overregulated genes in colon cancer (Figure 1), suggesting their importance in the establishment of tumoral processes [36]. In PAH transcriptomic analysis, AEBP1 and ZNF503 are both related to the negative regulation of transcription, and ZNF503 is related to the regulation of metabolism, cell proliferation, and epigenetic reprogramming. Moreover, in Table 2, there is an important number of PAH TFs involved in key biological processes like angiogenesis, positive and negative regulation of cell proliferation, cell proliferation, cell migration, cell differentiation, cell motility, cell activation, immune response, apoptosis, and epigenetics reprogramming that might be involved in the acquisition of hallmarks of cancer. Therefore, all are key to experimentally validate PAH as a model of inflammation-induced tumorigenesis, along with their target genes, according to the PAH GRN analysis (Table S1.3).

According to our transcriptomic analysis, CD has common overregulated genes with lung and colon cancer (Figure 1), located in the mitochondrion, extracellular exosome, membrane, receptor complex, and endoplasmic reticulum, related to metabolic pathways, ATP binding, and epigenetic reprograming (Table S1.10). ZNF3 is a transcriptional regulator of the zinc finger protein family, common in CD and colon cancer, that enables identical protein binding activity, and which might be involved in negative regulation of transcription by RNA polymerase II in cellular proliferation, migration, and invasion in colon cancer cells [158]. In CD transcriptomic analysis, ZNF3 was related to the negative regulation of transcription, the regulation of metabolism, cell differentiation, and epigenetic reprogramming. Moreover, in Table 3, there are an important number of CD TFs involved in key biological processes like metabolism, cell differentiation, cell proliferation, angiogenesis, apoptosis, cell development, cell migration, response to growth factor, response to cytokine, response to hormone, and epigenetics reprogramming that might be involved in the acquisition of hallmarks of cancer. Therefore, all are key to experimentally validate CD as a model of inflammation-induced tumorigenesis, along with their target genes, according to the CD GRN analysis (Table S1.6).

According to our transcriptomic analysis, UC has common overregulated genes with lung cancer (Figure 1), located in the mitochondrion, membrane, and extracellular exosome, related to multiple metabolic pathways, ATP binding, transit peptides, and epigenetic reprograming (Table S1.10). BZW2 is a transcriptional regulator common in UC and lung cancer, consistently co-expressed in a statistically significant manner in lung cancer [4,10] through glycolysis-mediated IDH3G lactylation, offering a theoretical basis for the targeted treatment of LUAD with glycolysis and HLA [159], and which shows local genetic variations of IBD effecting risk prediction in genome-wide association studies [160]. ZNF3 and BZW2 have not been previously related with IBD pathogenesis, and both were identified as common overregulated genes in colon and lung cancer, respectively (Figure 1), suggesting their importance in the establishment of tumoral processes. The association between IBD and gut cancer has been seen through the association between chronic intestinal inflammation and increased risk of colorectal cancer, small-bowel adenocarcinoma, cholangiocarcinoma, and anal cancer, as well as through an association between IBD treatment with chronic immunosuppression and the promotion of carcinogenesis [161]. In UC transcriptomic analysis, BZW2 is related to epigenetic reprograming. Moreover, in Table 4, there are an important number of UC TFs involved in key biological processes like metabolic process, circadian rhythm, cell differentiation, cell proliferation, cell development, response to lipids, response to hormones, and epigenetics reprogramming that might be involved in the acquisition of hallmarks of cancer. Therefore, all are key to experimentally validate UC as a model of inflammation-induced tumorigenesis, along with their target genes, according to the UC GRN analysis (Table S1.9).

### 4.4. Transcriptomic Regulation and Microbiota Interactions Within Inflammation and Tumorigenesis

In our transcriptomics analysis of the lung and gut, we have explained the role of the regulatory genes and membrane receptors in inflammatory and tumorigenic processes during host–microbiome crosstalk and added examples of how it could be contributing to these complex cellular processes, and the known interactions with microbiota and host membrane receptors that might be related to the activation of specific signaling pathways and regulatory programs. Bioinformatics-only studies are very powerful, analyzing complex scenarios like lung and gut inflammation-induced tumorigenesis mediated by microbiota signaling. However, they have inherent limitations that can impact the interpretation, accuracy, and applicability of their findings. The limitations come from the lack of experimental validation confirming the biological relevance and medical impact; therefore, the predictions remain as hypotheses. Bioinformatics-only studies must use high quality input data for the analyses, as errors, biases, or inconsistencies in raw data (e.g., sequencing errors, batch effects, incomplete datasets) can lead to misleading conclusions. Studies must use as many datasets as possible to avoid limited coverage, including all populations studied so far, and use all information available to account for individual variability, such as genetic heterogeneity or environmental influences (e.g., diet, microbiota, or stress). Several datasets including every inflammatory and tumoral disease were used for cross-independent dataset validation to ensure robustness and confirm consistency despite variability. Bioinformatics-only studies must also use bioinformatics tools able to yield reproductible results, computational algorithms capable of capturing as much complexity as possible, and dynamic characteristics like temporal changes, feedback loops, or adaptive responses of biological systems, correlating and integrating multiple omics analyses (e.g., genomics, transcriptomics, epigenomics, proteomics, metabolomics, and microbiomics).

To overcome the limitations of bioinformatics analysis and achieve a clear mechanistic explanation of how overexpressed genes in inflammatory diseases might be regulated by host–microbiome interactions and the association with the activation of specific signaling pathways that contribute to tumorigenesis, a structured approach is required. Therefore, our transcriptomic analysis will follow future experimental validations of inflammatory and tumorigenic microbiomes and transcriptomic regulatory networks of our population to (1) specify how the genes are upregulated (e.g., gene amplification, chromosomal translocation, epigenetic regulation (DNA methylation, histones modifications, and non-coding RNAs), and signaling dysregulation) during the inflammatory and tumorigenic process, providing context about the frequency and types of cancers where this overexpression is observed; (2) identify the downstream specific effect of every regulatory gene in expression (e.g., proliferation, survival, metabolism, invasion, and metastasis) mainly involved in the unlocking of phenotypic plasticity for the acquisition of the hallmarks of cancer; (3) identify the signaling pathways or networks affected by the gene’s overexpression; (4) address tissue-specific roles of the gene or variations in its oncogenic potential depending on the cellular context; and finally, (5) to highlight its implications for diagnosis, prognosis, or treatment.

Moreover, there are several knowledge gaps in host transcriptomic regulation and microbiota interactions within inflammatory diseases and tumorigenesis crucial for a deep understanding of how host genes are most susceptible to dysbiosis-induced changes, as well as how host genes regulate the formation and function of microbiome networks in health and disease. Future research must focus on the study of the following: (1) the composition, formation and function of microbiome networks in individuals and populations to identify a plausible global mechanisms linking microbiota and transcriptomic changes; (2) the influence of genetic polymorphisms, of host genes of transcriptional regulators and membrane receptors, in transcriptomic responses to microbiota and inflammatory triggers; (3) the temporal dynamics of transcriptomic changes during disease progression in response to microbiota shifts with single-cell and spatial transcriptomics to map localized host–microbiota interactions in inflamed or tumor-prone tissues; (4) the role of microbiota in regulating host coding (e.g., transcription and epigenetic factors) and non-coding RNAs (e.g., miRNAs, lncRNAs, and circRNAs), influencing inflammation and tumorigenesis; (5) the tumorigenic and inflammatory signaling pathways activated by host–microbial direct interaction through exosomes, membrane receptors, microbial metabolites (e.g., short-chain fatty acids) or pathogen-associated molecular patterns that selectively activate or repress transcriptional programs in host epithelial, immune (e.g., macrophages, T cells, and dendritic cells), and stromal cells in the tumor microenvironment; (6) the feedback loops between microbial metabolites, host transcription, and post-transcriptional modifications; and (7) the integration of transcriptomics with other omics data (e.g., genomics, proteomics, metabolomics, and microbiomics) to understand the multi-layered regulation of host–microbiota interactions in inflammatory and tumorigenic diseases, to finally develop microbiota-targeted therapies to beneficially influence host transcriptomic programs, such as probiotics, prebiotics, or microbiota transplantation, on host gene expression in inflamed or tumor-prone tissues. Therefore, to address these gaps, advanced technologies and experimental systems capable of replicating human microbiota and tissue microenvironments (e.g., single-cell high throughput sequencing, organoids, and gut-on-a-chip systems) must be used to provide deeper insights into these complex interactions.

Previous studies in lung and gut inflammation-induced tumorigenesis have used experimental models and advanced techniques to mimic chronic inflammation and specific pathogen infections to assess its impact on tumor development, including in vitro, in vivo, and clinical research approaches. We have previously made a review of studies that have used omics techniques (Genomics, transcriptomics, and epigenomics) to analyze the impact of host–microbiome interactions in inflammatory and tumorigenic diseases, reducing the analysis to the specific microorganisms, genes, signaling pathways, and epigenetic mechanisms involved [4]. The transcriptomic signatures generated by lung and gut inflammation-induced tumorigenesis models can be used to identify early-stage disease biomarkers involved in the molecular mechanisms of the diseases, enable precision diagnostics and targeted therapeutics, become prognostic markers capable of predicting disease outcomes by identifying expression patterns associated with progression or survival, can predict and monitor the response to advanced therapies in cancer patients, can allow the identification of new uses for existing drugs by correlating transcriptomic signatures with known drug mechanisms, can shape therapies to individual patients based on their unique gene expression profiles, and can help in designing vaccines by identifying immune-activating gene signatures after deep experimental validation. We propose an analysis paradigm, using transcriptomics as the first step and a central hub for integrating layers of data from genomics, epigenomics, proteomics, metabolomics, and microbiomics as DNA, RNA, and protein-interacting networks and complexes of human and microbiota crosstalk, involved in the regulation of inflammatory and tumorigenic disease mechanisms. Transcriptomics analysis has a profound impact on clinical applications, driving advances in diagnostics, prognostics, personalized medicine, and therapeutic development. As complex technologies and computational tools evolve, transcriptomics will become an increasingly integral part of clinical practice, enabling better outcomes for patients.

The observed association between lung and gut microbiota and PAH, CD, and UC phenotypes may arise from three scenarios. (1) Causation by dysbiosis: Dysbiosis could contribute to the pathogenesis of PAH, CD, and UC by mechanisms such as systemic inflammation, immune dysregulation, or altered metabolic profiles. The DEGs in common among the inflammatory disease sets show a significant association with the activation of these mechanisms, while the analysis of membrane receptors in rafts and complexes suggests that the microbiota may be the initial signal for their activation. (2) Consequence of inflammation: PAH, CD, and UC might themselves lead to changes in gut microbiota due to factors like hypoxia, altered hemodynamics, or medications. (3) Lastly, other shared risk factors, such as genetic predispositions, diet, lifestyle, or environmental factors might influence PAH, CD, and UC development, as well as lung and gut microbiota composition. Therefore, our experimental approach will seek to distinguish how, when, and where the dysbiosis of the microbiota is involved as a driver, a consequence, or merely present along with PAH, CD, or UC phenotypes.

## 5. Conclusions

The transcriptomic analysis of gut and lung inflammatory diseases highlights the impact of coding and non-coding regulatory RNAs in PAH (IRF9 and LINC01089), CD (ZNF91 and TP53TG1), and UC (ZNF91, VDR, DLEU1, SATB2-AS1, and TP53TG1) that might be related to the regulation of key membrane receptors on membrane rafts in PAH membrane receptors (CLN3, EFHD2, EFNB1, MYADM, NFAM1, PECAM1, PSEN2, and S1PR1), UC (ABCG2, CHP1, CXADR, DLG1, ERLIN2, HOOK1, PSEN1, RGMB, SLC6A4, and STX12), and a receptor complex in CD receptors (BMPR1A, ERBB2, FGFR3, GPR160, NTRK2, PPARG, PTPRF, VIPR1, and VDR), which interact with the characteristic inflammatory-related microbiome to control MAPK, NOTCH, PPAR, AMPK, and metabolic signaling pathways, as well as to unlock phenotypic plasticity for the acquisition of the hallmarks of cancer during lung and gut cell adaptation to inflammatory phenotypes. Our transcriptomic analysis suggests a complex crosstalk between the microbiome and inflammatory transcriptional regulatory network that participates in the same biological process. The annotation analysis and all experimental studies have demonstrated evidence that host–microbiome interactions through specific membrane receptors, and coding and non-coding RNAs identified as differentially expressed genes in inflammatory diseases, regulate gene expression in a specific spatial, temporal, and sequential manner during the inflammatory and possible subsequent tumorigenic process in the gut–lung axis. Lung and gut inflammatory and tumorigenic processes share important transcriptional regulators and the biological function of deregulated genes involved in the acquisition of the hallmarks of cancer, suggesting the significance of inflammatory diseases in gut and lung cell tumorigenesis. The microbiota seems to interact with specific receptors, playing a key role in the activation of key PAH, CD, and UC pathogenic signaling pathways, suggesting that targeting microbiota with probiotics or fecal microbiota transplantation might have a strong therapeutic potential in gut and lung inflammatory diseases.

PAH, CD, and UC are valuable models for studying inflammation-induced tumorigenesis because they sum up the interplay between chronic inflammation, tissue remodeling, immune dysregulation, and molecular alterations that underlie cancer development. Understanding these mechanisms can help to identify diagnostic and therapeutic targets for preventing and treating inflammation-induced tumorigenesis. There are possible mechanisms and indirect evidence that suggest an interplay between PAH and lung cancer, but direct causal links between PAH and lung cancer require further investigation through longitudinal studies to establish the incidence of lung cancer in PAH patients in every population, mechanistic studies exploring how specific molecular changes in PAH predispose lung tissue to tumorigenesis, and experimental validations of shared signaling pathways and biomarkers for PAH and lung cancer as we have identified to uncover new therapeutic targets and improve clinical management for at-risk patients. On the other hand, the association between IBD and gastrointestinal cancers is well established; however, more studies are needed to analyze the role of specific microbiome networks and their metabolites in tumorigenesis and differences in cancer risk among diverse populations and genetic backgrounds, where regular surveillance and early detection in IBD patients is critical for reducing cancer-related morbidity and mortality. To clarify these relationships, robust experimental studies, such as longitudinal cohorts or interventional trials (e.g., microbiota transplants or prebiotic/probiotic supplementation) are going to be a key part of our experimental validation of the microbiome and host transcriptomic regulatory network function in our populations.

## Figures and Tables

**Table 1:** Datasets analyzed for the transcriptomic analysis of gut and lung inflammatory diseases.

Dataset	Number of Samples
GSE15197	Normal (13) vs. Pulmonary arterial hypertension (18)
GSE117261	Normal (25) vs. Pulmonary arterial hypertension (58)
GSE113439	Normal (11) vs. Pulmonary arterial hypertension (15)
GSE256539	Normal (68) vs. Pulmonary arterial hypertension (152)
GSE53408	Normal (11) vs. Pulmonary arterial hypertension (12)
GSE6731	Normal (4) vs. Crohn’s disease (18)
GSE94648	Normal (22) vs. Crohn’s disease (50)
GSE207022	Normal (23) vs. Crohn’s disease (47)
GSE66407	Normal (99) vs. Crohn’s disease (103)
GSE83687	Normal (60) vs. Crohn’s disease (42)
GSE179285	Normal (31) vs. Crohn’s disease (47)
GSE186582	Normal (25) vs. Crohn’s disease (195)
GSE126124	Normal (21) vs. Crohn’s disease (37)
GSE102134	Normal (12) vs. Crohn’s disease (35)
GSE75214	Normal (22) vs. Crohn’s disease (58)
GSE59071	Normal (11) vs. Crohn’s disease (8)
GSE20881	Normal (73) vs. Crohn’s disease (99)
GSE6731	Normal (4) vs. Ulcerative colitis (9)
GSE83687	Normal (60) vs. Ulcerative colitis (32)
GSE66407	Normal (99) vs. Ulcerative colitis (161)
GSE179285	Normal (31) vs. Ulcerative colitis (23)
GSE126124	Normal (12) vs. Ulcerative colitis (7)
GSE94648	Normal (22) vs. Ulcerative colitis (25)
GSE36807	Normal (7) vs. Ulcerative colitis (15)
GSE11223	Normal (69) vs. Ulcerative colitis (63)
GSE10616	Normal (11) vs. Ulcerative colitis (10)
GSE92415	Normal (21) vs. Ulcerative colitis (87)
GSE48959	Normal (10) vs. Ulcerative colitis (9)
GSE47908	Normal (15) vs. Ulcerative colitis (20)

**Table 2:** PAH TFs involved in biological processes related to the acquisition of hallmarks of cancer.

Biological Process	Transcription Factors
Transcription	Positive: E2F4, FOXF1, FOXK1, GATA2, GLMP, HEY2, HIF3A, HOXA2, IRF9, MAF, RXRG, SOX7, TBX3, and ZNF335 Negative: AEBP1, BCL6, DACH1, FOXF1, FOXK1, GATA2, HEY2, HOXA2, KCNIP3, MAF, MIER2, SOX7, TBX3, and ZNF503 Complex: DACH1, E2F4, FOXF1, GATA2, HEY2, HIF3A, IRF9, MAF, RXRG, and TBX3
Metabolism	BCL6, DACH1, E2F4, FOXF1, FOXK1, GATA2, HEY2, HIF3A, IRF9, KCNIP3, MAF, RXRG, SOX7, ZNF333, ZNF335, ZNF362, and ZNF503
Angiogenesis	GATA2 and HIF3A.
Cell proliferation	Positive regulation: BCL6, FOXF1, GATA2, HEY2, and ZNF335. Negative regulation: BCL6, DACH1, GATA2, SOX7, and ZNF503.
Cell migration	DACH1, FOXF1, and GATA2.
Cell differentiation	BCL6, E2F4, FOXF1, FOXK1, GATA2, HEY2, HOXA2, MAF, ZNF335, and ZNF503.
Cell motility	DACH1, FOXF1, and GATA2.
Cell activation	BCL6, FOXF1, GATA2, and ZNF335.
Immune response	BCL6, FOXF1, GATA2, and ZNF335.
Apoptosis	BCL6, GATA2, HEY2, HIF3A, KCNIP3, SOX7, and ZNF346.
Epigenetics reprogramming	Phosphoprotein: BCL6, DACH1, E2F4, FOXK1, GATA2, IRF9, KCNIP3, MIER2, PHF1, TBX3, ZNF335, ZNF362, and ZNF503 Histone modifications: BCL6, GATA2, MIER2, and ZNF335. Histone deacetylase binding: HEY2 and MIER2. Epigenetic regulation of gene expression: ZNF335. Protein-DNA complexes: E2F4, FOXF1, FOXK1, HEY2, HIF3A, HOXA2, IRF9, KCNIP3, MAF, RXRG, SOX7, and TBX3.

**Table 3:** CD TFs involved in biological processes related to the acquisition of the hallmarks of cancer.

Biological Process	Transcription Factors
Transcription	Positive: FOXJ3, GPBP1L1, HINFP, KLF5, MLX, NFYC, OVOL1, OVOL2, PPARG, PPARGC1A, SMAD4, TFDP2, THRB, UBP1, VDR, ZNF91, ZNF395, and ZNF600. Negative: ARHGAP35, FOXN3, HINFP, ID3, KLF5, MIER3, MLX, OVOL1, OVOL2, PPARG, SMAD4, TFDP2, THRB, UBP1, VDR, ZFP3, ZNF3, ZNF91, and ZNF124. Complex: KLF5, MLX, NFYC, PPARG, SMAD4, TFDP2, THRB, and VDR.
Metabolism	ARHGAP35, FOXJ3, HINFP, ID3, ISX, KLF5, MLX, NFYC, OVOL1, OVOL2, PPARG, PPARGC1A, SMAD4, UBP1, VDR, ZFP3, ZNF3, ZNF395, ZNF600, and ZNF704
Cell differentiation	ARHGAP35, HINFP, ID3, KLF5, OVOL2, PPARG, PPARGC1A, ROGDI, SMAD4, VDR, and ZNF3.
Cell proliferation	KLF5, OVOL1, OVOL2, PPARG, PPARGC1A, ROGDI, SMAD4, and VDR.
Angiogenesis	KLF5, OVOL2, PPARG, and UBP1.
Apoptosis	ID3, PPARG, PPARGC1A, SMAD4, and VDR.
Cell development	ARHGAP35, KLF5, OVOL2, PPARG, and SMAD4.
Cell migration	ARHGAP35, OVOL2, PPARG, and PPARGC1A.
Response to growth factor	OVOL2, PPARGC1A, and SMAD4.
Response to cytokine	KLF5, PPARG, PPARGC1A, and SMAD4.
Response to hormone	PPARG, PPARGC1A, and VDR.
Epigenetics reprogramming	Phosphoprotein: ARHGAP35, FOXJ3, FOXN3, GPBP1L1, MIER3, MLX, OVOL2, PPARG, PPARGC1A, SMAD4, TFDP2, TSHZ1, UBP1, ZNF3, ZNF395, and ZNF704. Acetylation: PPARGC1A, ROGDI, SMAD4, and TFDP2. Protein phosphorylation: PPARGC1A, and SMAD4. Protein-DNA complexes: FOXJ3, FOXN3, ISX, KLF5, MLX, NFYC, PPARG, PPARGC1A, SMAD4, TFDP2, THRB, TSHZ1, UBP1, and VDR.

**Table 4:** UC TFs involved in biological processes related to the acquisition of the hallmarks of cancer.

Biological Process	Transcription Factors
Transcription	Positive: ELF4, FOXJ3, HINFP, GPBP1L1, HNF4A, HNF4G, HOXA2, HOXA5, KLF5, MLX, MLXIP, NFYC, NR1I2, NR1H4, NR3C2, NR5A2, OVOL2, PPARA, PPARGC1A, PPARGC1B, SATB2, THRA, VDR, ZNF91, ZNF112, ZNF219, and ZNF395. Negative: ARHGAP35, CBFA2T2, HINFP, HNF4A, HOXA2, KLF5, MLX, MNX1, MXI1, NR1H4, NR1I2, OVOL2, PPARA, PPARGC1B, SATB2, THRA, VDR, ZFP3, ZNF91, ZNF124, ZNF219, ZNF253, ZNF439, and ZNF350. Complex: HNF4G, KLF5, MLX, MLXIP, MXI1, NFYC, NR1I2, NR5A2, SATB2, THRA, VDR, and ZNF350. Coregulator binding: HNF4A, NR1H4, NR3C2, NR5A2, and PPARA.
Metabolism	ARHGAP35, CBFA2T2, ELF4, FOXJ3, HINFP, HNF4A, HNF4G, KLF5, MLX, MLXIP, MXI1, NFYC, NR1I2, NR3C2, NR5A2, OVOL2, PPARA, PPARGC1A, PPARGC1B, THRA, VDR, ZFP3, ZKSCAN1, ZNF124, ZNF350, ZNF395, ZNF44, ZNF813, and ZNF823.
Circadian rhythm	HNF4A, PPARA, and PPARGC1A.
Cell differentiation	ARHGAP35, CBFA2T2, HINFP, HNF4A, HOXA2, KLF5, NR5A2, OVOL2, PPARA, PPARGC1A, PPARGC1B, THRA, and VDR.
Cell proliferation	HNF4A, KLF5, MXI1, NR5A2, OVOL2, PPARGC1A, and VDR.
Cell development	ARHGAP35, CBFA2T2, HNF4A, HOXA2, KLF5, OVOL2, and PPARA.
Response to lipid	HNF4A, HNF4G, HOXA2, NR1I2, NR3C2, NR5A2, PPARA, PPARGC1A, PPARGC1B, THRA, and VDR.
Response to hormone	HNF4A, HNF4G, NR1I2, NR3C2, NR5A2, PPARA, PPARGC1A, PPARGC1B, THRA, and VDR.
Epigenetics reprogramming	Phosphoprotein: ARHGAP35, BZW2, CBFA2T2, ELF4, FOXJ3, GPBP1L1, HNF4A, HNF4G, LRRFIP2, MLX, MLXIP, MNX1, NR1H4, NR3C2, OVOL2, PPARGC1A, PPARGC1B, SATB2, ZKSCAN1, ZNF219, and ZNF395. Acetylation: BZW2, HNF4A, MLXIP, MNX1, NR1H4, and PPARGC1A.

## Data Availability

The transcriptomics datasets analyzed in this study can be found in the GEODasets repository of the NIH. The original contributions presented in the study are included in the article/Supplementary Material; further inquiries can be directed to the corresponding author.

## Supplementary Materials

The following supporting information can be downloaded at https://www.mdpi.com/article/10.3390/cells14010001/s1: Table S1: Pulmonary arterial hypertension (PAH), Crohn’s disease (CD), Ulcerative Colitis (UC) DEG selection, annotation, and coding and non-coding regulatory networks; S1.1: PAH upregulated DEG selection and David’s annotation analysis of common upregulated DEGs; S1.2: PAH ToppFun annotation analysis of common upregulated DEGs; S1.3: PAH cncTRN and GRN analysis; S1.4: CD upregulated DEG selection and David’s annotation analysis of common upregulated DEGs; S1.5: CD ToppFun annotation analysis of common upregulated DEGs; S1.6: CD cncTRN and GRN analysis; S1.7: UC upregulated DEG selection and David’s annotation analysis of common upregulated DEGs; S1.8: UC ToppFun annotation analysis of common upregulated DEGs; S1.9: UC cncTRN and GRN analysis; S1.10: Common inflammatory and tumorigenic DEG analysis.
